# Oil/Water Mixtures and Emulsions Separation Methods—An Overview

**DOI:** 10.3390/ma16062503

**Published:** 2023-03-21

**Authors:** Maria Helena José, João Paulo Canejo, Maria Helena Godinho

**Affiliations:** CENIMAT|i3N, Department of Materials Science, School of Science and Technology, NOVA University Lisbon and CEMOP/UNINOVA, 2829-516 Caparica, Portugal

**Keywords:** oil-adsorption materials, oil-absorption materials, oil removing, water removing, oil microdroplets, oil/water emulsions, oil/water separation

## Abstract

Catastrophic oil spill accidents, oily industrial wastewater, and other types of uncontrolled release of oils into the environment are major global issues since they threaten marine ecosystems and lead to a big economic impact. It can also affect the public health of communities near the polluted area. This review addresses the different types of oil collecting methods. The focus of this work will be on the different approaches to materials and technologies for oil/water separation, with a special focus on water/oil emulsion separation. Emulsified oil/water mixtures are extremely stable dispersions being, therefore, more difficult to separate as the size of the droplets in the emulsion decreases. Oil-absorbent materials, such as sponges, foams, nanoparticles, and aerogels, can be adjusted to have both hydrophobic and oleophilic wettability while displaying a porous structure. This can be advantageous for targeting oil spills in large-scale environmental and catastrophic sets since these materials can easily absorb oil. Oil adsorbent materials, for example, meshes, textiles, membranes, and clays, involve the capture of the oily material to the surface of the adsorbent material, additionally attracting more attention than other technologies by being low-cost and easy to manufacture.

## 1. Introduction

### 1.1. From Biomass Fuels to a Fossil Fuel Economy

Since the discovery of fire, wood fuels and other types of biomass fuels have been used to obtain heat, light, and other forms of energy. Over the years, with the increase in population, the advances in technology, and the increasing necessity for power motivated the search for a more efficient fuel. Although, even with the advances in agriculture, solar-based energy generation, and other types of renewable energy sources, such as wind and waterpower generation, the power supply was still limited and the search for an even better alternative was inevitable [1]. The turning point in the major transition from biomass fuels to fossil fuels was marked by the historic invention of the steam engine and the consequent industrial revolution era [2]. At this point, the cost of wood fuel increased significantly. The harvesting of forest trees began to be regulated, and the power efficiency needed at this point was incompatible with these types of fuels. Moreover, in the 18th century, coal was used as a substitute for biomass fuels and, ultimately, in the 19th century was marked by the additional introduction of natural gas, petroleum, and its derivatives, thus beginning the oil age era with the invention of internal combustion engines [1].

Despite all the advantages that come from this type of fuel, problems have arisen with its use associated with its extraction and distribution, as is the example of oil spills [3]. Water treatment is a very important topic nowadays, and several works are reported in the literature that are concerned with the removal of zinc ions (Zn^2+^) [4] and other harmful substances [5].

### 1.2. Oil Spills—An Overview

Between 1965 and the 1970s, global oil consumption was, on average, 22,042 terawatt-hours (TWh) per year. That sums to a capacity of about 1.895 × 10^9^ tons of oil equivalent (TOE), in which an average of 635,000 tons of oil per year were accidentally spilled into the ocean [6].

Nowadays, when addressing recent worldwide oil consumption, from the years 2010 to 2018, the average global oil consumption was approximately 51,500 TWh per year, equivalent to approximately 4.428 × 10^9^ TOE [6]. With this number in mind, just in the last decade (the 2010s), the average oil spill lost was around 164,000 tons.

In 2019, four large oil spills were recorded in North America, South Asia, and Brazil, which resulted in approximately 3500 tons of oil spilled into the ocean due to problems with oil tankers, such as collisions, equipment failure, and explosions [7].

Besides oil spillage, oily effluent discharges, such as oil−water emulsions from a wide range of industries, for instance, from the automobile industry, food industry, the textile industry, the refineries, as well as from oil explorations, are all major sources of water pollution and contamination [8]. These events resulted in catastrophic damage to the ecosystem and human health, not only as a consequence of the oil spills or discharges themselves but also as a consequence of the inherent cleaning processes [9]. Additionally, the death of surface sea organisms, the death by asphyxiation of many fish, birds, turtles, etc., the destruction and death of corals, and finally, the exposure of organisms and humans to some toxins, directly and indirectly through our food chain, are some examples of the hazard that this situation can cause to the ecosphere [3]. Thus, it is of extreme importance to continue the study for efficient oil capture methods, for innovative wastewater treatment, and to explore new methods and techniques, primarily for oil/water emulsion separation.

### 1.3. Oil/Water Conventional Separation Technologies

**Figure 1:**
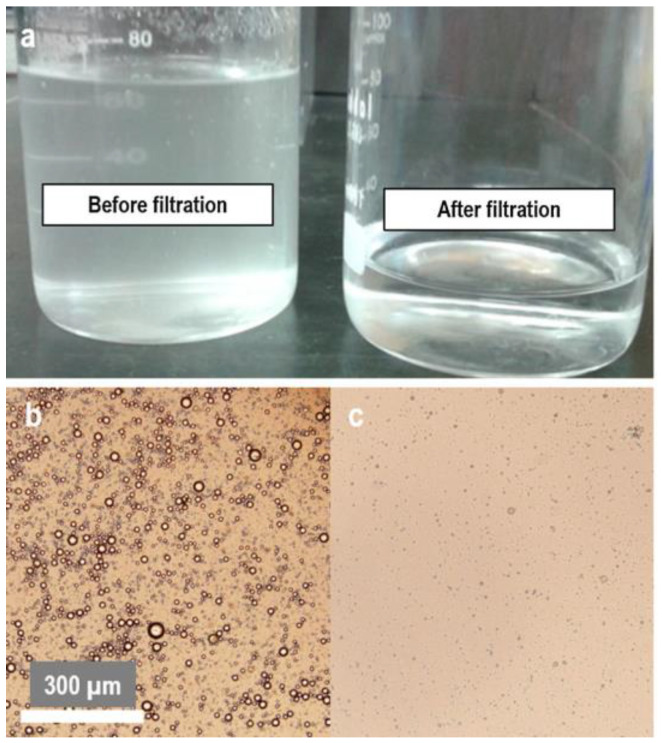
(**a**) Photo of an oil/water emulsion; (**b**) Optical microscopy image of an oil/water emulsion before filtration. (**c**) Optical microscopy image of an oil/water emulsion after filtration. Adapted by permission from Springer Nature: Springer, Cellulose, All-cellulose composite membranes for oil microdroplet collection, Ana P. C. Almeida, João Oliveira, Susete N. Fernandes, Maria H. Godinho, João P. Canejo [Copyright] (2020) [3].

Micrometer droplets have their origin in emulsified oily water [10], as seen in Figure 1, as an after-effect of oil spills or oily industrial wastewater.

These are an outcome of economic and technological growth and are a consequence of a water/oil emulsion, in which the originated microdroplets have, generally, dimensions of 20 μm or less, being undetectable to the naked eye and being unable to be captured by the conventional methods, as mentioned below [11]. In the last years, one of the most used conventional techniques in water/oil separation has been air flotation [12], a physical treatment that consists of removing impurities in water using air under pressure. This forms bubbles that will cause the impurities to float to the surface, allowing them to be separated from the water. Other methods are gravity separation [13], adsorption [14], sedimentation by centrifugation, which consists of rotating the solution by centrifugal force favoring the sedimentation of the solution compounds [15], and biological treatment [16] in which microorganisms are introduced to eliminate the organic parts and stabilize pollutant compounds of the oily wastewater [8,17]. However, these processes face major challenges, such as high costs, high complexity, low efficiency, and, more importantly, incompatibility with collecting oil microdroplets [18].

### 1.4. Microdroplets Removal Technologies

Currently, one of the most commonly employed physical techniques for microdroplet collection is electroflotation [8], used to treat oily wastewater and to efficiently collect microsized droplets. It consists of generating hydrogen and oxygen bubbles on the electrode surface while water electrolysis is simultaneously occurring. These gas bubbles will then interact with the oil droplets, which will cause the oil/gas bubbles to rise. Oil has a lower density compared to water, so it tends to rise to the surface so that it can be easily removed by employing either the skimming method, where floating oil and oil emulsions are removed by a device at the surface [19]. Electrocoagulation [20] is a similar process that is described by the dissolution of a sacrificial anode, the result of applying a difference in potential between the electrodes. One of the resulting products is flocs, remains from the sacrificial anode, in the form of metallic hydroxides, within the wastewater to be treated that is capable of adsorbing its impurities, such as oil, even oil microdroplets from oil/water emulsions [21]. Hydroxyl ions formed on the cathode [21,22] that are also released to the wastewater form a precipitate with the pollutant particles and, hence, flocculation. This sludge can be removed either by floatation or sedimentation [23]. However, these approaches have limitations, for instance, high material requirements and high machinery costs, together with high energy consumption [20].

### 1.5. Nanotechnology

Nanotechnology is an emerging area that has been constantly evolving and revolutionizing different fields of science such as environmental engineering [24], medicine [25], electronics [26], chemistry [27], and many more [28].

Richard Feynman introduced, for the first time, the concept of nanotechnology when addressing a new perspective for miniaturization coupled with the idea of atomic material manipulation. This important mark happened in 1959, in his famous lecture “There’s plenty of room at the bottom”, at the annual session of the American Physical Society at Caltech [29].

Over the years, significant changes happened, and nowadays, nanotechnology can be defined as the modern science and engineering involved in the design, synthesis, characterization, and application of materials at the nanoscale, defined between 1 and 100 nanometers (nm) [30]. Here, physical, chemical, and biological properties of nanomaterials, like optical properties, reactivity, and thermal stability, change in comparison to the bulk material properties [30]. Features such as quantum phenomena and the relation between the decrease in particle size and the consequent increase in surface area are responsible for a change in some of the properties of the materials [30].

Nanomaterials can be classified by their morphology being, for example, nanorods, nanowires, nanocrystals, nanowiskers, and others, or by their dimension, in which nanoparticles and quantum dots fit into the zero-dimensional category, while nanowires and nanorods are labeled as one-dimensional. In two-dimensional nanosheets and three-dimensional materials like nanocrystals, nanomaterials are made up of multiple layers [31].

#### Nanoparticles

As already stated, nanoparticles are zero-dimensional nanostructures. They can be classified according to their morphology and chemical and physical characteristics being divided into carbon-based, metal, ceramic, semiconductor polymeric, or lipid-based nanoparticles [32].

Nanoparticles (NPs) have countless applications in different areas of science. Such things can be justified by their ability to be used as mechanical reinforcement of matrices (structural), by their high surface area, and by their size influence on the optical, electrical, chemical, and physical properties of a substance [32]. All these properties, and mainly its high surface-to-mass ratio, make them interesting for use on the surface of a material. In the case of environmental applications, NPs have a very alluring role in treating contaminated water since contaminants like oil can be easily absorbed by some nanoparticles [32], such as magnetite nanoparticles [33], cellulose nanocrystals [34], and others [35].

### 1.6. Oil/Water Separation Technologies

#### 1.6.1. Existing Methods for Oil/Water Separation

The current global situation regarding the removal and treatment of residues from water sources is becoming an environmental concern that can no longer be avoided or ignored [36]. The ecosystem is being greatly harmed as a consequence of polluters such as oil spills and oily residues present in wastewater. Therefore, to address this issue, water treatment has recently gained much attention from researchers worldwide, whose focus is to find new, more efficient, and cost-effective materials and removal technologies that present low toxicity, good recyclability, and are easy to handle [36].

#### 1.6.2. Oil or Water Absorbent Materials

Some sorbent materials appear as an asset for oil removal in high-scale environmental catastrophes since they can easily absorb the oil, meaning that the oil is integrated into the absorbent material without the release of residues into the ecosphere [37]. For efficient oil collection, the chosen materials must be hydrophobic and oleophilic, porous, and present good oil selectivity. Some sponges, gels, particles, and foams are examples of materials that present these mentioned abilities and, thus, are among the most frequently studied porous oil sorbent materials in our days [38].

##### Foams and Sponges

**Figure 2:**
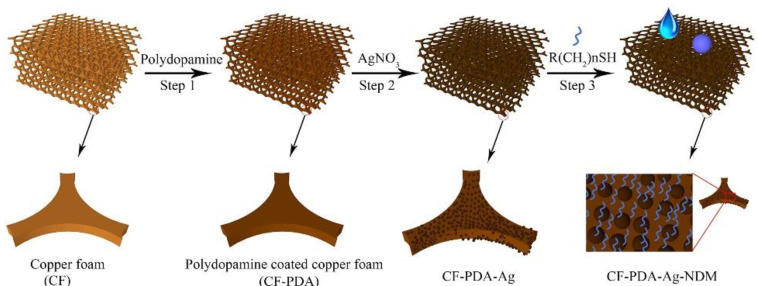
Fabrication of a superhydrophobic surface using copper foam as a substrate. Reprinted from Applied Surface Science, Vol 413, Wei Zhou, Guangji Li, Liying Wang, Zhifeng Chen, Yinlei Lin, “A facile method for the fabrication of a superhydrophobic polydopamine-coated copper foam for oil/water separation”, Pages No.140, Copyright (2017), with permission from Elsevier [42].

Foams and sponges are 3D materials, which are characterized by their high surface area, elevated porosity, low density, high absorption rate, and being extremely lightweight [39]. By definition, foam is a solid or liquid substance where gas bubbles, just like air, are trapped inside, and a sponge is a porous material that can be made from various materials, such as ceramic, metallic, or even polymeric materials [40]. These assets, along with their ability to quickly absorb some substances such as certain pollutant materials and, when squeezed, to release the collected material, constitute a great advantage when collecting oil from big environmental sets [41]. Additionally, some of them display the capability to absorb oils at a high rate of up to 140 g/g (gram of oil per gram of sorbent) [41]. Certain foams and sponges can also demonstrate high durability and great mechanical strength due to the ability to use and adjust pre-existing foam and sponge structures for oil separation purposes [41]. One example of the preeminent structural materials being studied for oil/water separation purposes, regarding its performance and customization, is copper foam (CF). Regarding this subject, an already bought commercial CF was surface modified with polydopamine, AgNO_3_, and n-dodecyl mercaptan and studied at each stage by Zhou et al. [42], see Figure 2.

This surface modification allowed the transformation of a hydrophilic foam into a final superhydrophobic foam, having shown to separate different types of oils, such as hexane, sesame oil, and octane, in an efficient manner, higher than 95%, displaying high-performance cycles up to 30 uses, where a cycle is one separation efficiency test [42]. Polymer foams are also an efficient and cheap alternative, demonstrating good reusability [38]. The most common primal matter used in polymer-based foam production are polyurethane (PU) and melamine (ME) since they are cost-effective, easy to produce, and abundant [38]. For instance, polyurethane (PU) sponge is a light weight polymer that demonstrates high porosity, high oil absorption capacity, cost effectiveness, and the ability to be produced at a large scale [43]. Liu et al. [43] bought a low-cost commercial PU sponge that had magnetic properties due to Fe_3_O_4_ nanoparticle coating. The result was a sponge that presented superhydrophobic and superoleophilic wettability. It was proven to be efficient in harsh environments, such as salty water or very alkaline or acidic solutions, displaying an absorption capacity of up to 35 times its own weight, valid for either light or heavy-density oils [43]. Apart from that, the magnetic properties allow for easy collection of the sponge from the environment [43]. In the same way, Wu et al. [44] manufactured a low-cost and flexible polymer-based graphene foam (PGB) by a low-cost self-assembly technique of graphene sheets on a PU skeleton, which was both superhydrophobic and superoleophilic and capable of collecting different oil types, while being efficient, above 90% efficiency, and reusable after 300 utilization cycles. Moreover, its unique 3D structure, presenting a more wrinkled structure than a standard PU foam, allows the foam to exhibit great mechanical properties, even when bent, compressed, or twisted [44]. In conclusion, in addition to all the advantages mentioned above, both sponges and foams also possess the huge benefit of being easily customizable with different material coatings, depending on their specific purpose, in order to finally achieve efficient oil/water separations.

##### Aerogels

Aerogels are a type of material that can be used in applications related to oil/water separation [45]. They are 3D materials, which are produced by sol−gel synthesis, that consists, briefly explained, of the formation of a gel, a rigid and highly porous network, originating from reactions taking place between colloidal particles in solution, sol [46]. The sol-gel technique has the potential to be used in many areas. As an example, sol−gel is reported for the production of nano-ZnO coatings that can be used as protection for corrosion and anti-icing applications [47].

This 3D network, for oil/water separation purposes, can function as a sponge, exhibiting, however, higher flexibilities. They are also characterized by their high surface areas, elevated porosity, and low density [48]. A sponge-like aerogel with those properties was produced by Yu et al. [48] by a sol−gel method. In order to achieve hydrophobicity abilities, organoalkoxysilanes were chosen as precursors [48]. The final aerogel demonstrated good absorption capacities up to 10 cycles. In the same manner, Bo et al. [49] studied a hydrophobic BiOBr-silicone aerogel, fabricated by a sol−gel method, that was able to separate the oil from water, as well as to degrade, by photolytic degradation, the pollutant oil [49]. Aerogels can be immersed into oily wastewaters, just like foams and sponges, but they also can be integrated into a gravity separation process [14].

##### Nanoparticle Incorporation

Certain nanoparticles have an important task in oily water treatments since oil can be easily photodegraded, absorbed, or trapped at the surface of the nanoparticles due to characteristics such as its high surface area to mass ratio and oil selectivity [32]. Nanoparticles can contribute excellent mechanical, electrical, optical, and morphological properties when used complementarily to customize previously made structures [32]. Gupta et al. [38] highlighted in their work that the use of NPs can positively contribute to material composites, increasing their surface roughness and having the ability to help increase the underwater hydrophobicity and oleophilicity properties of the material [38]. This is again justified by some properties associated with nanoparticles, such as their high surface area and their size influence on the chemical and physical properties of a substance [32].

Ge et al. [50] mentioned, in their work, the use of a previously bought polyurethane sponge that was coated by the dip-coating method with polyfluorowax and hydrophobic silica nanoparticles (HPS) intended to establish a superhydrophobic wettability behavior to the material. It has been demonstrated to maintain its absorption capacity for up to 10 cycles [50].

**Figure 3:**
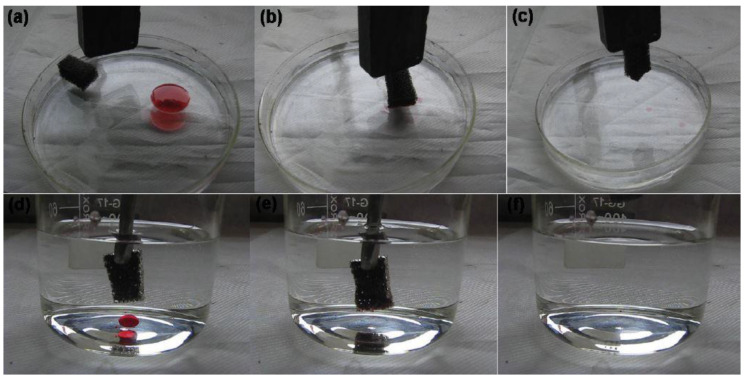
Demonstration images for the removal of hexadecane oil, dyed with red, from the water surface using a piece of foam controlled by a magnet (**a**–**c**); Reprinted from Colloids and Surfaces A: Physicochemical and Engineering Aspects, Bo Ge, Xiaotao Zhu, Yong Li, Xuehu Men, Peilong Li, Zhaozhu Zhanga, Versatile fabrication of magnetic superhydrophobic foams and application for oil/water separation, Pages No.4 Copyright (2015), with permission from Elsevier [50].

Magnetic nanoparticles can be attractive not only for the cleaning process but also for manipulating the foam and removing it from the water after the separation process. For instance, Ge et al. [50], in their work, functionalized a previously existing polyurethane foam with magnetic superhydrophobic nanoparticles of Fe_3_O_4_ to capture oil from oily wastewater. The foam showed a magnetic behavior due to the addition of the Fe_3_O_4_NPs and had the consequent ability to be easily manipulated toward polluted areas with a magnet, as shown in Figure 3. It also showed an oil absorption capacity of up to 70 g/g, a good compressing resistance of up to 200 cycles, and the possibility to scale its production in order to clean larger areas [50].

#### 1.6.3. Oil or Water Adsorbent Materials

Adsorbent materials are another type of technology for oil recovery and oil/water emulsions separation. This material category involves the attachment of the collecting material to the surface of the adsorbent material and stands out from other technologies by being low-cost, easily fabricated, and ideal for applications mainly in industrial wastewater treatments. This category includes separation materials such as meshes, filters, films, clays, and membranes [38].

##### Meshes

The utilization of meshes became a very alluring technique for wastewater separation purposes; this is justified by their easy and cheap manufacture, for example, by 3D printing [51], and by their high availability and low cost when commercially purchased [52]. They also have excellent efficient separation rates justified by their large pores of about 50 µm, being, therefore, capable of separating large volumes of oil from water at low pressures and using a gravity-driven separation method [53]. Typically, meshes are made from metals, such as stainless steel or aluminum, and in order to make these materials viable for oil/water separation, the mesh surface needs to be customized to favor the adjustment of its wettability. For this purpose, materials such as nanoparticles, oxides, and polymers are being studied [38,54].

**Figure 4:**
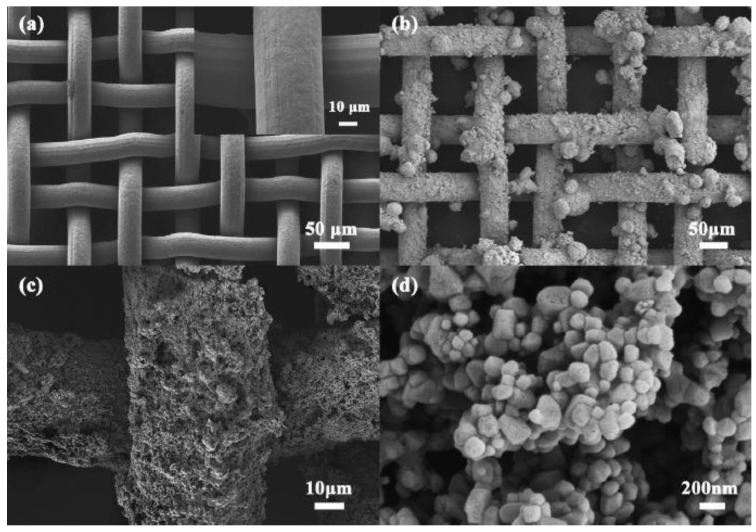
SEM images of (**a**) the original stainless-steel mesh and (**b**–**d**) the TiO_2_-coated mesh surface at low and high magnifications, respectively. Reprinted from Colloids and Surfaces A: Physicochemical and Engineering Aspects, Volume 489, Jian Li, Long Yan, Wenfang Hu, Dianming Li, Fei Zha, Ziqiang Lei, Facile fabrication of underwater superoleophobic TiO_2_ coated mesh for highly efficient oil/water separation, Pages No. 3, Copyright (2016), with permission from Elsevier [52].

Li et al. [52] produced an underwater, superoleophobic, TiO_2_ nanoparticle-coated stainless steel mesh, represented in Figure 4. The stainless-steel mesh, which was previously commercially achieved, was coated, by spray-coating, with TiO_2_ NPs and polyurethane (PU), both providing an increase in the binding forces between the NPs and the mesh. Because of its water affinity, the oil is captured by the mesh surface, giving rise to a 99% separation efficiency and demonstrating a reusability of over 40 separation cycles, having also confirmed a high resistance to fouling, which occurs when the oil droplets are squeezed, using high pressure, into the membrane, thus blocking the pores of the membrane [52]. Wang et al. [55] also manufactured an environmentally friendly MnO_2_ nanocrystal-coated stainless steel mesh, previously purchased and then coated with the MnO_2_ nanocrystals by hydrothermal synthesis. The final mesh showed underwater superoleophobicity behavior, concentrating the oil at the coated mesh surface and, consequently, demonstrating good separation selectivity, with efficiencies of around 95.6% [55]. As another example, cellulosic meshes can be used, being more advantageous in terms of cost-effectiveness and environmentally friendliness than other methods displayed in the literature. Koh et al. [51] printed 3D cellulose acetate meshes, which demonstrated anti-fouling properties and an oleophobic nature, that was able to achieve 95% separation efficiencies. Also, the printed meshes were capable of “self-cleaning”, that is, by immersing the contaminated mesh in water, the previously collected oil droplets detached themselves from the mesh, being capable of coagulating into larger droplets [51]. This 3D printing methodology allows a higher control of the mesh design [51], which is difficult to achieve with already purchased meshes, and cellulose acetate presents a more environmentally friendly alternative to the typical stainless steel meshes due to its biodegradability [56].

##### Clays

Clays, like montmorillonite and bentonite, are currently being studied for oil sorbent applications, such as oil spills and wastewater treatments, as a consequence of their high oil adsorption capacity [57]. These materials can be easily used as composite material and are known to improve both physical and chemical properties of the composite, such as enhancing the mechanical properties while reducing the materials’ flammability [58]. Wang et al. [59] studied the incorporation of montmorillonite (MMT) and polyvinylidone (PVP), a porogen, on an ultrafiltration membrane of poly(vinylidene fluoride) (PVDF), produced by the phase inversion method [59]. MMT was proven to enhance, when together with the PVP, the hydrophilicity of the membrane, and it also changed its surface roughness. In this report, it was concluded that the membrane showed the potential to remove pollutants from wastewater [59]; however, they did not present the results for oily wastewater separation. Polymers have the advantage of efficiently dispersing clay particles [60] and, considering that fact, Kahraman et al. [57] produced a hydrophobic and oleophilic electrospun polyacrylonitrile (PAN) membrane with Cloisite 30B, an organoclay, which was incorporated onto the nanofibers. It was concluded that by adding 3% of clays to the nanofibers, the oil adsorption capacity increased by 160 times its own weight, having a value of around 180 g/g. This was mostly justified by the increase in hydrophobicity caused by the clay layers. Comparing these results to pure PAN membranes, which are naturally hydrophilic, the previous results clearly did not display oil absorption capacity [57].

##### Textiles

**Figure 5:**
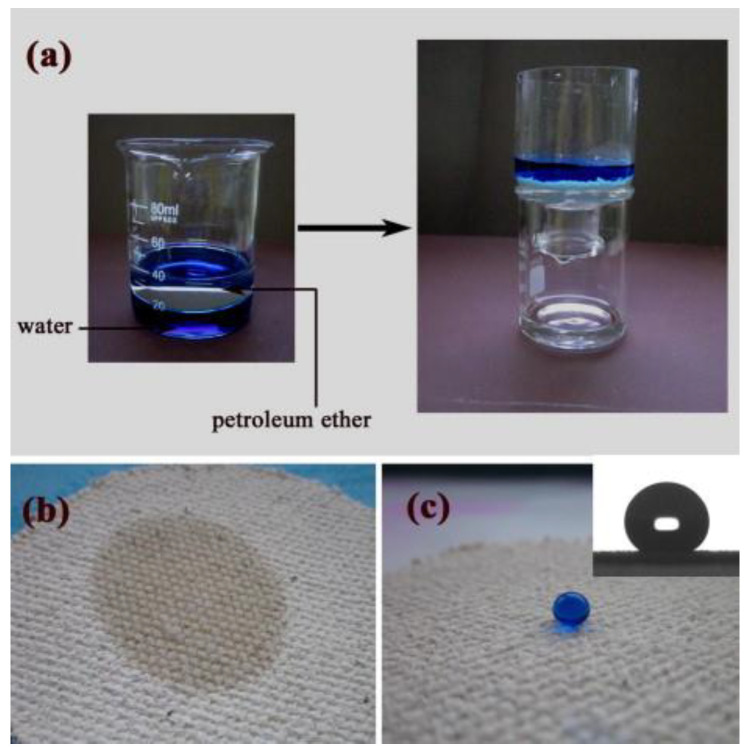
(**a**) A mixture of water (dyed with blue for easy observation) and petroleum ether was on the fabric surface, petroleum ether passed through the surface, and the water was held on the surface. (**b**) Spreading of petroleum ether on the coating surface. (**c**) After 3 min, the petroleum ether volatilized completely, and the coating recovered to its superhydrophobicity. Reprinted from Chemical Engineering Journal, Volume 231, Xia Zhang, Tie Geng, Yonggang Guo, Zhijun Zhang, Pingyu Zhang, Facile fabrication of stable superhydrophobic SiO_2_/polystyrene coating and separation of liquids with different surface tension, Pages No. 414, Copyright (2013), with permission from Elsevier [64].

Textiles are lightweight materials and a more flexible alternative when compared to other technologies, such as metallic meshes. Furthermore, some of them can demonstrate high adsorption rates and good mechanical strength even under harsh environments [61]. However, the textile surface needs to be functionalized in order to possess selectivity abilities making them capable of being only used for water removal, presenting hydrophilicity and oleophobicity, or an oil-removal type, demonstrating hydrophobicity and oleophilicity [62]. Zhang et al. [62] reported the production of a flexible and uniformly coated trichloromethylsilane polyester textile, a superoleophilic textile that demonstrated reusability and good oil/water separation efficiency since almost no water could be perceived. However, they did not present efficiency results since they claim to be difficult to attain because of the volatility of octane oil [62]. In the same manner, Xue et al. [63] used a commercially available poly(ethylene terephthalate) (PET) textile, coated, by immersion, with a silica hydrophobic sol−gel that provided an increase in roughness of the textile surface. These demonstrated superhydrophobic and superoleophilic behavior, and a high and rapid affinity with the crude oil used in the oil/water mixture. However, after the textile immersion in the oil/water mixture, it was found some leftover crude oil in the tin, thus showing a poor oil separation efficiency [63]. In another work, Zhang et al. [64] also used silica to produce highly mechanically stable SiO_2_/polystyrene (PS), nanocomposite-coated fabrics. The fabrics were purchased from local textile stores and were made from 70% cotton and 30% polyester. For the coating, the fabrics were immersed in the SiO_2_/PS suspension for approximately two minutes [64]. Contrary to the results demonstrated by Xue et al. [63] work results, this final fabric displayed excellent durability, good flexibility, and an excellent oil/water separation efficiency of around 97%, see Figure 5 [64].

##### Membrane Technology for Oil and Oil Microdroplets Collection

Membrane technologies arose as a way to overcome the gaps and disadvantages present in the traditional oil/water removal methods previously mentioned; these include expensive costs, high complexity, and low oil/water separation efficiencies. Industries are the number one beneficiary of this technique as a consequence of its simple production and easy manipulation. Additionally, these membranes show the ability to easily separate oil/water emulsions due to their small pore sizes [65]. Their mechanism of operation resembles the selective barrier of transport between two distinct phases, being those, in this case, the oil phase and the water phase [66].

One of the most common membrane methods used in industries comprises pressure-driven membranes [36,66], which can be categorized by their pore sizes. Microfiltration (MF) membranes present higher fluxes than nanofiltration (NF) membranes and ultrafiltration (UF) membranes, showing, however, a higher probability to foul [67]. Ultrafiltration membranes possess smaller pore sizes than all the MF membranes, being capable of removing substances of smaller sizes. They do, however, display poor antifouling properties [68]. Nanofiltration membranes, based on a thin membrane of 0.1 to 0.2 µm in thickness, having pores ranging between diameters of 2 to 5 nm, and in which separation is primarily based on electrostatic repulsion and size exclusion, demonstrate that they can easily permeate oily solutions [69,70], or even the reverse osmosis (RO)membrane where the pressure exerted on the fluid flux is inversely proportional to the pressure on the layer surface [71].

Membranes can also be divided according to their primary components, polymers and inorganic materials, the most commonly studied materials for developing membranes [72]. Inorganic membranes, on the one hand, normally derived from ceramic materials, such as silica, alumina, or titania, when compared to other membranes, demonstrate greater temperature resistance and good mechanical properties. Unfortunately, their use translates into greater fabrication complexity and higher costs [73]. On the other hand, polymeric membranes are very cost-effective and easy to produce. They display, however, rapid deterioration and have a diminished flux, justified by their tendency to foul [20,74].

**Figure 6:**
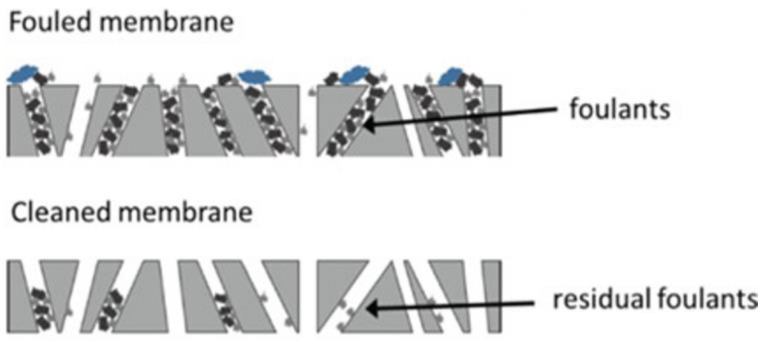
Schematic of a fouled membrane. Reproduced from [76].

In order to avoid fouling, whose mechanism of operation is represented in Figure 6, the surface of the membrane can be functionalized, thus increasing its hydrophilicity, by, for example, decreasing the oil adhesion to the surface and, therefore, reducing the probability of the membrane to foul [72]. Nanofibers can be used as a valuable solution in overcoming this issue, displaying a threadlike fiber structure with fiber lengths ranging from 50 to 300 nanometers [75]. For that reason, they will be highlighted in the following chapter in the form of an overview of different types of nanofiber membrane material properties and developments in this area of study.

### 1.7. Advantages in the Use of Nanofibers in Oil/Water Separation

#### Nanofibers

**Figure 7:**
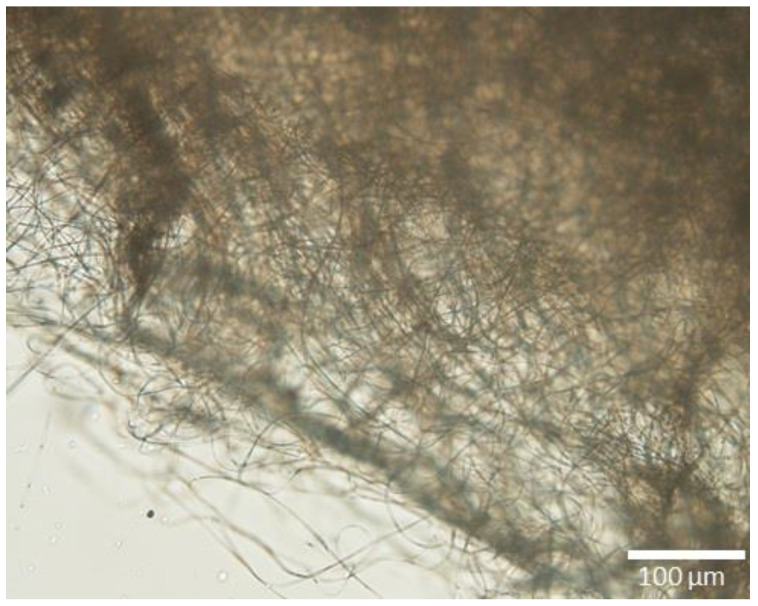
Optical microscope image of electrospun cellulose acetate nanofibers.

Nanofibers (NFs), which are represented in Figure 7 by an optical microscopy image displaying their morphology, have already been employed in numerous applications focusing on different types of water treatments [77] and have become alluring for applications in oil/water removal, due to their easy customization when attaining a membrane format.

This trait allows for the use of a variety of different types of material, leading to different fiber diameters/dimensions and to the possibility of producing different levels of permeability, surface area, and porosity [78].

##### Nanofiber Fabrication Methods

Some of the most employed nanofiber fabrication techniques include mechanical force methods, such as extrusion [75], which consist of extruding one or even two polymers simultaneously from a single hole, making it possible to have different types of fiber rearrangements and designs [75]. Aside from this technique, phase separation can also be used to produce nanofiber matrixes, relying on the physical incompatibility between a polymer dissolution and a solvent after a gelation process followed by dehydration of the matrix [79]. Meltblowing is another nanofiber production technology, which consists of a one-step methodology that comprises the melting and consequent extrusion of thermoplastic polymers, such as polyester, polystyrene, and polyethylene, producing, therefore, micro and nanofibers [79].

**Figure 8:**
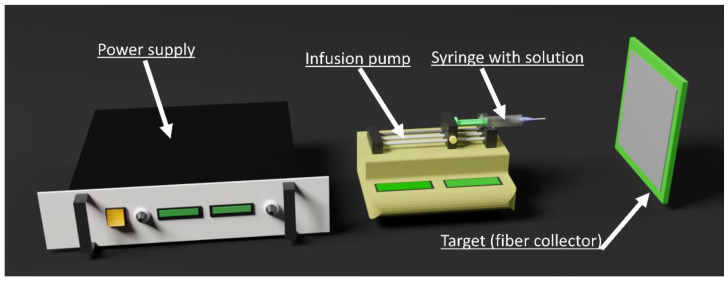
Schematic of a typical electrospinning setup.

Finally, one of the most commonly used methods regarding nanofiber production is electrospinning, represented in Figure 8 in its most traditionally used setup, which is a method based on electrostatic forces [80]. This technique became an attractive, cheap, and widespread technology since it allows an ability to have a certain control of the final non-woven mats, for example, its porosity, its thickness, and its morphology [81]. Most importantly, this technique allows choosing from a vast panoply of materials, although polymer materials are the base material, depending on the final intent. In this case, for oil/water separation, choosing the right type of material can produce a membrane with high porosity, elevated surface area, high flexibility, and good water or oil permeability [82], which are all important features, since, as already mentioned, wettability of the membrane plays a crucial role on the filtration process [83]. In electrospinning, several parameters can influence the properties of the nanofibers. These can be divided into three main categories: processing conditions, where variables such as flow rate can be highlighted; applied voltage, a factor that has control upon the resultant fiber diameter [84]; and the diameter of the needle, target geometry, and the distance from the tip of the needle to the target [80]. Others are solution parameters, such as solvent evaporation rate [85,86], solution viscosity [80,87], surface tension parameter [61,88], and finally, ambient parameters where the most important conditions are temperature [89,90] and humidity [91].

##### Nanofiber Membranes for Oil/Water Separation

The use of nanofibrous membranes is a quick and environmentally friendly alternative to air flotation, centrifugation, and other traditional but more complex methods. This method is greatly advantageous due to its high surface areas, its proven efficient oil/water separation, its cost effectiveness, its simplicity of manufacture and operation, its easy manipulation, and the possibility of customization of its wetting properties [55].

**Figure 9:**
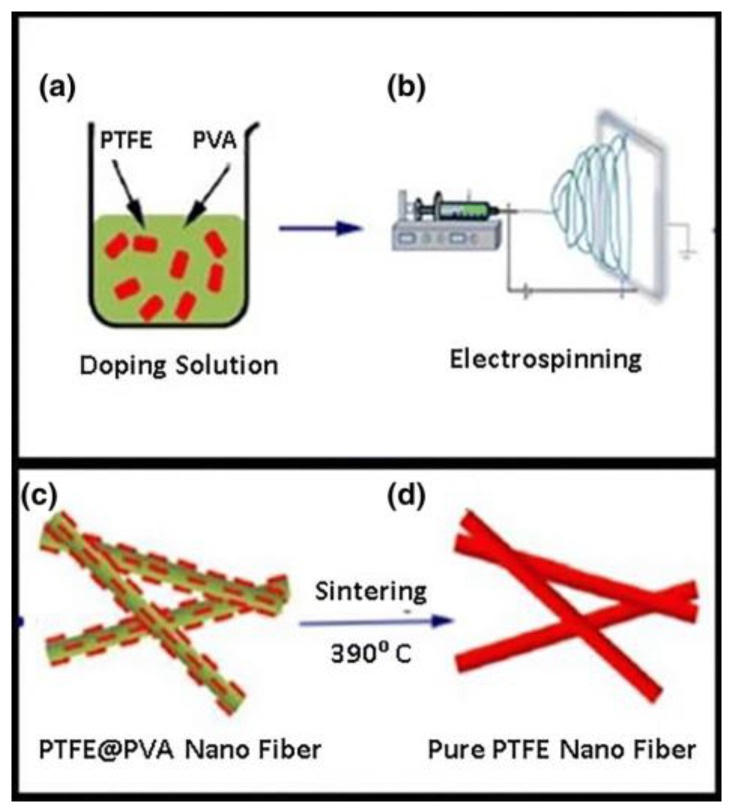
Manufacturing procedure of the PTFE nanofibrous membrane. Used from [92].

When referring to wettability, these membranes can be divided into two main types, the oil removing ones and the water removing ones [83]. Oil removing membranes are characterized by their hydrophobicity and oleophilicity, meaning that, for example, in a gravity-driven separation method, they can repel water and easily adsorb and filter the oil phase [55]. Patel et al. [92] proposed, in their work, an oil removing electrospun polytetrafluoroethylene (PTFE) thin and porous membrane for ultrafiltration purposes (see Figure 9). Using the latter, oil would flow under capillary separation, and water would be repelled [92]. This work demonstrated it was cheap to produce due to electrospinning and showed an efficiency of oil/water separation of around 99% for oil/water separation [92]. Also, He et al. [88] presented, in their study, a poly(arylene ether nitrile) (PEN) nanofibrous membrane, produced by electrospinning and hot-pressing techniques, that can be potentially used for oil/water emulsion separations in harsh environments. This conclusion arises from the displayed efficiency separation ratio of 99% after 24 h of use [88]. The biggest disadvantage allied to this type of membrane is membrane fouling, which reduces their efficiency of separation and, posteriorly, leads to their deterioration [36].

The use of water removing membranes is described in the literature [93] and represents a better alternative to oil removing ones since fouling is almost impossible. These membranes function through water absorption and filtration, together with the surface retention of the oil. In water removing techniques, cellulose acetate, chitosan, some polymeric or ceramic nanoparticles, and other materials are used to obtain a membrane that is both superhydrophilic and superoleophobic [55]. Obaid et al. [36] presented, in their work, the production of a water removing electrospun polysulfone (PSf) membrane immersed in a NaOH solution in order to obtain a membrane with underwater superoleophobicity and with a high-water flux property, which can overcome fouling. Aside from the ability to not suffer fouling, this membrane also displayed an oil/water separation efficiency of roughly 99.99% [36].

Membrane categorization can also be evaluated in terms of their primal material, being carbon-based, ceramic-based, and polymer-based nanofibrous membranes [94]. Polymer-based nanofiber membranes arose from the need to find a cost-effective alternative, which also displayed great flexibility and ease of handling [94]. For instance, Hong et al. [95] produced, in their study, an electrospun cellulose acetate nanofibrous membrane with high chemical stability, being resistant to extreme conditions, namely to very acidic or alkaline environments [95]. It is important to highlight that this is normally one of the greatest difficulties and disadvantages allied to this type of membrane [95]. The developed membrane also demonstrated superoleophobicity and superhydrophilicity, having a high separation flux and, consequently, a high separation efficiency of more than 99%, even in harsh environments and for more than one cycle of oil/water separation [95]. The author suggests that this membrane has the ability to be used in large-scale oil/water separations. Even though the membrane presents high flux and chemical stability in harsh environments, they did not suggest a methodology to implement it on larger scales than a laboratory gravity separation setup.

Ceramic-based nanofibrous membrane fabrication recently became very attractive, taking into consideration that they are typically low-cost materials in which the membrane demonstrates inertness, together with high chemical, thermal, and mechanical stability, making them ideal when treating harsh and corrosive environments [96]. Typically, ceramic materials chosen for this type of membrane could be silica, zirconia, zeolite, alumina, and other oxides [96]. Titanium dioxide, TiO_2_, a semiconductor that demonstrates to be non-toxic, photocatalytic, and water immiscible, is one of the most used oxides for this purpose [97]. Zhang et al. [98] manufactured in their work, by an easy sol-gel electrospinning and polymerization method, titanium dioxide nanofibrous membranes incorporated with TiO_2_ nanoparticles, proven to demonstrate high flexibility and chemical stability, nontoxicity, and good porosity [98]. The membranes also displayed high photocatalytic activity, capable of eliminating organic pollutants from water [98].

When comparing ceramic-based membranes with polymer-based ones, the first outshine the latter when performing in certain aggressive environments, such as highly acidic or highly alkaline means, since they do not deteriorate nor suffer modifications to their structure, making them suitable for oil/water separation in almost every environment [96]. Nevertheless, these membranes demonstrate the occasional occurrence of fouling, which can be explained by limitations regarding the pore sizes of the membranes. Barbosa et al. [99] produced in their work, by the secondary growth method, a very effective hydrophilic and oleophobic zeolite membrane for oil/water separation purposes [99]. In their study, when comparing the alumina comprising zeolite membrane with the one which did not have the zeolite crystals, some oil clogging was observed in the latter membrane. This is justified by the formation of an oil layer at the surface on both membranes, resulting in a better oil removal percentage. In spite of this fact, the zeolite crystals membrane showed a better efficiency, explained by its high porosity, in comparison to the low porosity of the ceramic-based one [99].

**Figure 10:**
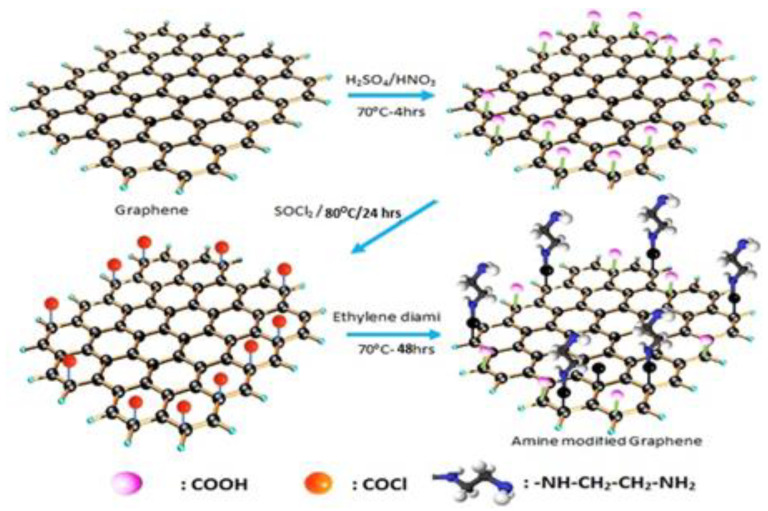
Different steps involved in the amine functionalisation of xGnP. Reprinted from Water Research, Volume 103, J.A. Prince, S. Bhuvana, V. Anbharasi, N. Ayyanar, K.V.K. Boodhoo, G. Singh, Ultra-wetting graphene-based PES ultrafiltration membrane—A novel approach for successful oil-water separation, Pages No. 311, Copyright (2016), with permission from Elsevier. [102].

Finally, carbon-based membranes are the last main category that exists, and they stand out from the previous ones due to their high chemical and mechanical stability and easy regeneration, which means that after physical or chemical cleaning, the membrane still presents the efficiencies that are presented in the beginning—high surface area and antifouling abilities [94]. Carbon-based membranes are characterized by having a wettability that is intrinsically oleophobic and hydrophilic. Concerning their composition, they are primarily composed of carbon nanotubes or graphene as the main material [100]. One of the most preeminent ongoing studies regarding the use of carbon nanotubes is their incorporation into polymer-based membranes, where, due to the alignment of the nanotubes, the water flux more than duplicates as a consequence of the surface smoothness of the nanotube walls [101]. Furthermore, this arrangement of the carbon nanotubes creates an additional porous layer, or stack of layers, in which its pore size can influence the collection of oil emulsion droplets of smaller diameters [101]. Graphene, when applied to a polymer membrane matrix, has also demonstrated improvement in the performance of the overall system. For instance, Prince et al. produced, in their work, a graphene-based PES membrane (see Figure 10), proving that graphene can enhance, by 43%, the PES membrane wettability [102].

The summary of the oil removal efficiency for the works cited can be observed in Table 1 for oil/water mixture separation.

### 1.8. Oil/Water Emulsion Separation

Concerning oil/water separation, one of the most recent adversities faced by industries, as well as the scientific community, is the difficulty in collecting oil from oil/water emulsions [103]. Oil pollution is still a problem, affecting our health and environment in current times, prominently oil/water emulsions, justified by the higher difficulty inherent in the collection of such small oil droplets [9].

**Figure 11:**
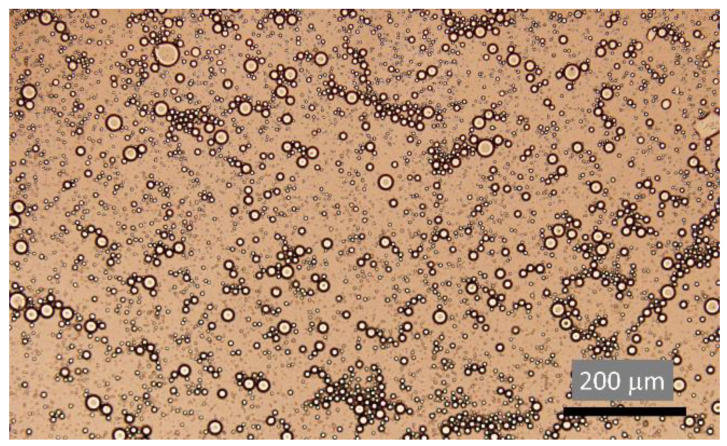
Optical image of oil/water emulsion observed in optical microscope.

Emulsified oil/water mixtures, as represented by an optical image of emulsions in Figure 11, are extremely stable dispersions [103] since they present a highly strong oil/water interface that becomes more difficult to separate as the size of the droplets in the emulsion decreases [104,105]. Oil microdroplets are a product of these emulsions and are categorized by having dimensions smaller than 20 μm, therefore, presenting themselves as a great obstacle since they are unable to be captured by conventional methods, which were described in earlier chapters [12]. Lately, and as already mentioned in Section 1.4, the most commonly used techniques, such as electroflotation [10] and electrocoagulation [22], have restraints, such as high material requirement, expensive machinery together with high energy consumption [22], which justify the need to continue the study for more effective solutions, possibly by combining different types of wettability with the right porous substrate [106].

#### 1.8.1. Oil or Water Absorbent Materials

##### Foams

Foams, as mentioned before, are 3D materials characterized by their low density, lightweight, and high surface area, also having the great capability to absorb liquid substances, depending on their wettability [41]. Wang et al. [107] in their work studied a cost-effective and eco-friendly method using a purchased polyurethane (PU) foam that was posteriorly coated in an alkaline medium, which contained dopamine, dodecanethiol, and fly ash. Fly ash is a fine waste powder derived from burning fossil fuels and municipal waste. This substance not only has proven to have an important role in emulsion separation, it also contributes to reducing the environmental burden consequent on the burning of these substances [107]. The foam proved to have superhydrophobicity, even under harsh mediums (very salty, acidic, or alkaline) for 15 cycles. Additionally, it displayed the ability to separate oil/water emulsions with efficiencies of about 93% [107]. Yang et al. [108] prepared, in their study, a commercial Ti foam with a one-step femtosecond laser, whose function was to treat the surface to be superhydrophilic and underwater superoleophobic, achieving separation efficiencies of 99% even in oil/water emulsion separations [108]. In the same manner, Luo et al. [109] also purchased a commercial titanium (Ti) foam that was after anodized in non-toxic fluorine containing electrolyte, which is the same as introducing ˗O ˗Ti ˗F groups into the Ti foam, forming a superhydrophilic foam that have been demonstrated to be ideal to separate oil/water emulsions. After the emulsion separation tests, it demonstrated a 99% of oil/water emulsion separation efficiency as well as anticorrosive properties [109]. Ti-based materials have once again demonstrated high potential for water treatments, mainly due to their nontoxicity and chemical stability. As for carbon foams, Yang et al. [110] produced a foam, in their work, fabricated by carbonization of a 3D, commercially available, melamine foam, that was compressed afterwards. The foam compression was made in order to avoid a possible foam collapse, and to increase its mechanical properties. This foam presented underwater superoleophobicity and underoil superhydrophobicity, proved to have excellent oil/water emulsion separation efficiencies of higher than 98%, and demonstrated environmental stability even in harsh environments [110].

##### Aerogels

**Figure 12:**
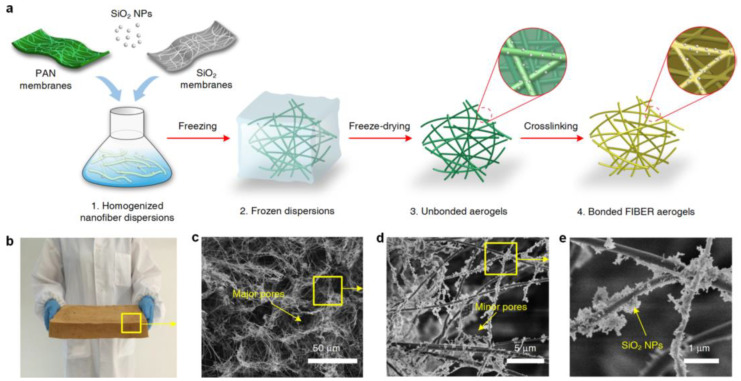
(**a**) Schematic showing the synthetic aerogel steps; (**b**) Optical image of fiber aerogel on a large scale of 2.5 L; (**c**–**e**) Microscopic architecture of fiber aerogels at various magnifications; Y. Si et al., “Superelastic and Superhydrophobic Nanofiber-Assembled Cellular Aerogels for Effective Separation of Oil/Water Emulsions”, ACS Nano, vol. 9, no. 4, pp. 3791–3799, 2015, https://doi.org/10.1021/nn506633b. Copyright (2015) American Chemical Society [111].

Aerogels, as seen in Figure 12, are highly porous 3D materials that can behave as sponges while displaying high surface areas, low density, and good flexibility [48].

Si et al. [111] reported, in their work, a flexible and superhydrophobic nanofiber-assembled cellular aerogel fabricated by deposition of electrospun nanofibers made of SiO_2_ and polyacrylonitrile (PAN) and having SiO_2_ nanoparticles incorporated in them, originating a 3D assembly, that was after freeze-dried to obtain the aerogel. The final product resulted in a structure with very low density, high recyclability, and capable of efficiently separating oil/water emulsions by gravity-driven separation with about 99% efficiency, while using a simple gravity separation method and presenting antifouling properties [111]. Moreover, Yue et al. [112] presented, in their study, a lightweight and superhydrophobic aerogel derived from wastepaper and banana peels, which was produced by the combination of freezing cast, freeze drying, and pyrolysis methodologies. The separation was effectuated by means of a gravity-driven method, concluding that the aerogel presented an absorption capacity of 35 to 115 times its own weight, and, even for oil/water emulsion separation, it achieved separation efficiencies up to 99.6% [112]. In a final example, Chaudhary et al. [113] produced, in their work, a non-toxic, hydrophilic, and highly porous chitosan and agarose-based aerogel; a polysaccharide was used as a pore formation and coating agent. The result was an eco-friendly membrane, presenting biodegradability, and displaying a separation efficiency of 99%, which indicates the potential of this membrane to be used in industrial settings. Summarizing, aerogels can easily separate oil/water and oil/water emulsions, making use of rather simple separation methods and resulting in high separation efficiencies, with the additional advantage of having a tendency to display antifouling properties.

#### 1.8.2. Oil or Water Adsorbent Materials

##### Membranes

Membrane filtration has played an important role in the oil/water emulsion separation subject, as they present favorable properties in the collection of such small oil droplets, a consequence of their ability to achieve smaller membrane pore sizes [114]. Furthermore, electrospun membranes demonstrate, typically, a high surface area, showing the ability to have control of the nanofibers’ size and the possibility to incorporate active chemistry into the network [115]. However, there are still drawbacks to overcome since, with smaller pore sizes, the probability of membrane fouling increases [105]. Zhou et al. [73] report, in their work, the fabrication of a hydrophilic membrane, in which hydrophilicity decreases the oil adhesion, therefore, reducing the probability of fouling. This ceramic microfiltration membrane was made from a commercially available Al_2_O_3_ membrane, which was modified with a ZrO_2_ coating by hydrolysis of ZrCl_4_, making the membrane more hydrophilic. These membranes proved to have an oil rejection above 97.8% [73].

**Figure 13:**
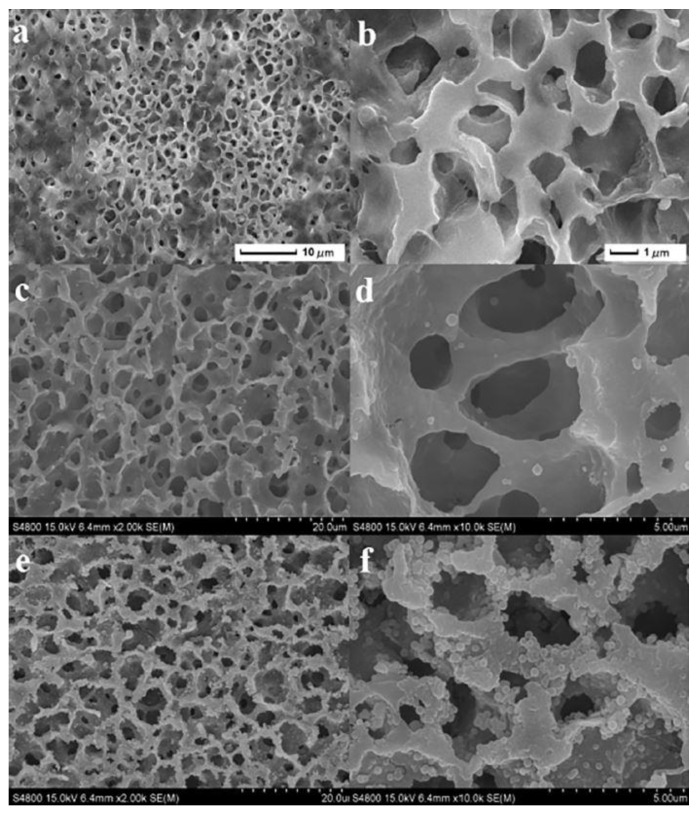
SEM images of (**a**,**b**) a simple PVDF membrane, (**c**,**d**) a PVDF membrane with pDA coating, and (**e**,**f**) the final membrane with SiO_2_ nanoparticles added to the final membrane surface. Reprinted from Separation and Purification Technology, Volume 209, Jiuyun Cui, Zhiping Zhou, Atian Xie, Minjia Meng, Yanhua Cui, Siwei Liu, Jian Lu, Shi Zhou, Yongsheng Yan, Hongjun Dong, Bio-inspired fabrication of a superhydrophilic nanocomposite membrane based on surface modification of SiO_2_ anchored by polydopamine towards effective oil-water emulsions separation, Pages No. 4, Copyright (2018), with permission from Elsevier [116].

Cui et al. [116] produced, in their study, using a phase-inversion process, a SiO_2_ nanocomposite polyvinylidene fluoride membrane (PVDF), which displayed resistance to fouling, see Figure 13. SiO_2_ nanoparticles proved to create roughness at the surface of the membrane and showed a decrease in its pore size. Regarding wettability, the membrane exhibited superhydrophilicity and superoleophobicity, as well as a high separation efficiency, approximately 98%, when collecting oil/water emulsions even after 10 cycles [116]. This eco-friendly membrane also showed resistance to fouling and an ability to regenerate, meaning that, after a separation cycle, the membrane can be recovered and reused, by simply washing it with ethanol and water [116]. Zhang et al. [117] mentioned, in their work, the successful production of a polymer-based nanofiber membrane with superhydrophobic and superoleophilic properties, making use of a commercially available polyester fabric, which was dip-coated in a solution containing a polymer of intrinsic microporosity (PIM-1) and fluorinated alkylsilane (PTES). It was also capable of removing oil from an oil/water mixture and oil from oil/water emulsions, with oil droplets diameters ranging from 10 to 30 µm, displaying separation efficiencies rounding 99.95% and 99.97% respectively, for up to 30 cycles [117]. Correspondingly, Almeida and coworkers [9] produced a cheap and environmentally friendly cellulose acetate (CA) electrospun composite membrane with cellulose nanocrystals (CNC) stamped by screenprinting on the surface of the membrane. It successfully demonstrated a separation efficiency of 83% for oil/water emulsions containing dispersed microdroplets, with diameters ranging from 10 to 15 µm, using gravity force filtration. Obaid et al. [118] manufactured, in another work, an electrospun Polyvinylidene fluoride (PVDF) nanofibrous membrane that was posteriorly modified with Triethylamine to avoid fouling [118]. PVDF is commonly used in applications for treating and filtrating different types of wastewater. The result was a superhydrophilic and superoleophobic membrane with high separation flux, and an efficiency of 99% for different types of oil and oil/water emulsions, having the potential to be easily implemented in industries for water treatment purposes [118].

##### Clays

**Figure 14:**
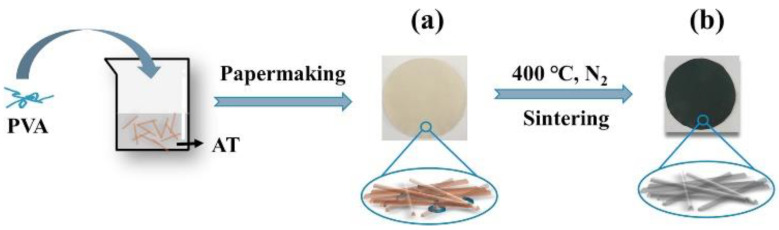
Schematic illustration of the preparation process for AT-based NFMs. (**a**) Unsintered AT-based NFMs, (**b**) Sintered AT-based NFMs. Reprinted from Ceramics International, Volume 43, Yekai Zhu, Dajun Chen, Novel clay-based nanofibrous membranes for effective oil/water emulsion separation, Pages No. 9465, Copyright (2017), with permission from Elsevier [119].

Clays have been shown to improve the mechanical strength and wettability of the filtration material [40,119] being also cost-effective, abundant in nature, and easy to integrate into membranes [70]. Due to that and their high adsorption capacity, clays have shown a high potential for oil/water emulsion wastewater treatment. In order to prove that, Mota et al. [120] used, as part of their study, modified Brazilian clays (green calcium bentonite−aluminum clay minerals) to analyze further their performance in oil/water emulsion separation. Clay powders were modified with surfactants into their interlayer space, becoming organoclays, in order to achieve hydrophobicity and increase their adsorption capacity for pollutants such as oils. The modified clays were weighed with 0.5 g of each sample and dispersed into 50 mL solutions containing oil/water emulsions. The clays displayed adsorption capacities up to 9.7 g/g, presenting oil removal efficiencies of up to 96% [120]. Moazed et al. [121] used, in their study, a combination of bentonite organoclay with anthracite in oil/water emulsions and were able to attain oil removal efficiencies up to 98%, and proved to have a quick sorption rate after 1 h of testing [121], proving therefore to be a good solution to treat oily wastewater emulsions. Finally, and employing clays into polymeric NFs, Zhu et al. [122] produced, in their work, a low-cost and hydrophilic ultrafiltration with attapulgite, a natural clay, and poly(vinyl alcohol) (PVA) nanofibrous membrane, via papermaking and posterior sintering technology (see Figure 14).

The membrane showed it had small pore sizes, about 12 nm, and the potential to be used for oil/water emulsion since it demonstrated a separation efficiency of around 97%, as well as good antifouling properties [122]. However, the authors obtained inferior efficiencies for higher separation pressures, which can be explained by the penetration of smaller oil microdroplets into the membrane [122]. After all, it can be concluded that clays display good oil adsorption capacities, mostly when treated with surfactants in order to show hydrophobicity. Furthermore, when used as a composite, they have proven to improve the chemical and physical properties of the matrix material, constituting, therefore, an interesting area of study.

**Figure 15:**
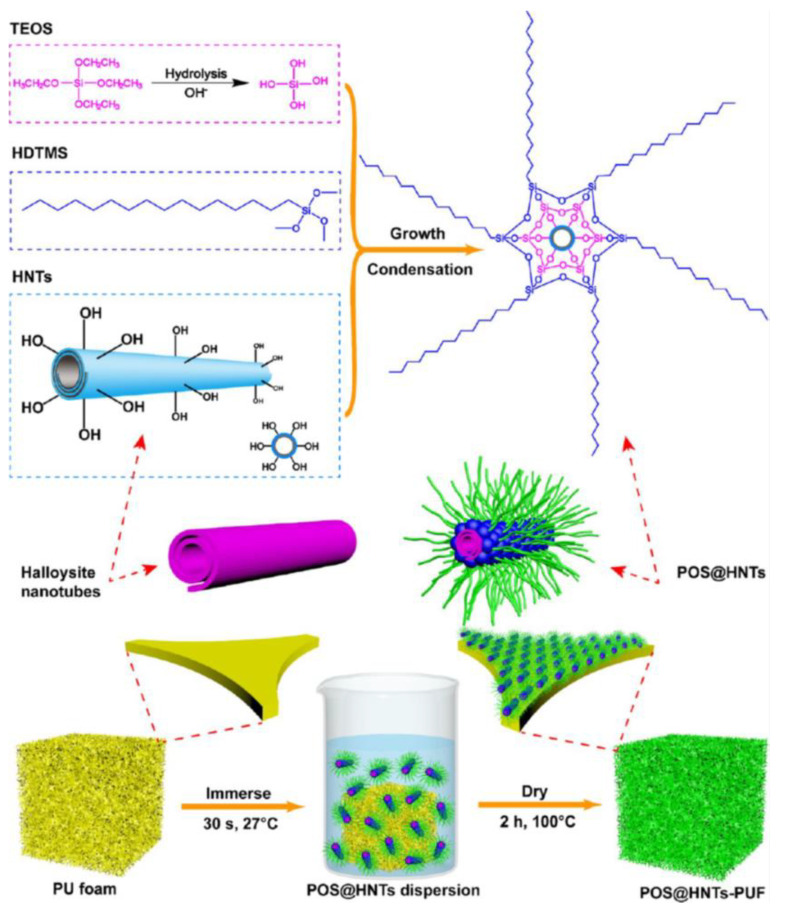
Schematic showing the preparation and reaction mechanism of the superhydrophobic coated polyurethane foam Reprinted with permission from F. Wu, K. Pickett, A. Panchal, M. Liu, and Y. Lvov, “Superhydrophobic Polyurethane Foam Coated with Polysiloxane-Modified Clay Nanotubes for Efficient and Recyclable Oil Absorption”, ACS Appl. Mater. Interfaces, vol. 11, no. 28, pp. 25445–25456, July 2019, https://doi.org/10.1021/acsami.9b08023. Copyright (2019) American Chemical Society [124].

Also reported in the literature is the use of clay nanotubes for oil/water separation [123,124,125], with results that show the positive potential of its use. As an example, Wu et al. [124] report on polymer foam prepared by dip coating in silanized clay nanotube dispersion (see Figure 15). The produced foam shows drastically enhanced lipophilicity, oil absorption (105 times its weight in chloroform), flame retardancy, and a capability of maintaining its properties during 10 cycles.

The use of halloysite clay nanotubes is reported as a stabilizer for oil/water Pickering emulsions [125] and as a way to favor the growth of bacteria that promote the degradation of the oil [126]. Nano clays can also be used as an emulsion stabilizer when functionalized with amphiphilic polypeptoids [123].

Due to their potential, it is suggested that research teams further endure the study of clays for applications in this area.

The summary of the oil removal efficiency for the works cited can be observed in Table 2 for oil/water emulsion separation.

## 2. Conclusions and Future Perspectives

Within the absorbent materials category, it is crucial that they present an adequate wettability, typically, both superhydrophobic and superoleophilic behavior, a great absorption ability, good selectivity, and, finally, a highly porous structure. Foams and sponges are extremely lightweight and of low density, with 3D porous structures having the potential to be introduced and directly used, for example, at the oil spill site. Beyond the high range of materials that can be used in these kinds of applications, there are polymers, such as polyurethane, and metals, i.e., copper, which display the advantage of being commercially available and of easy posterior modification. They present oil/water separation efficiencies of around 95% and a good mechanical strength to compression, from 30 to 300 cycles. As for oil/water emulsions, they also have been demonstrated to be a good separation method. Polyurethane, titanium, and carbon foams were observed to have successfully separated both phases, with efficiencies rounding 93 to 99%. These characteristics make them one of the technologies of choice for oil/water separation in case of environmental disasters or vast oily wastewater spots. In the same way, aerogels are also highly porous networks that possess a high surface, low density, and good flexibility. They can be made from silicone, chitosan, and even from biomass, such as banana peels and paper waste. They are normally produced through sol−gel synthesis and have the tendency to be manufactured to display an oleophilic wettability. However, since they can be employed as a filter to separate oil/water mixtures and emulsions by means of gravity separation, they can also be hydrophilic. As a result, separation efficiencies rounded 99%, even for oil/water emulsions. As for nanoparticle incorporation, these have the advantage of not only being highly absorbent but also of providing other properties to the composite materials. For example, Fe_3_O_4_ NPs provide magnetic properties to the composite, while silica NP incorporation provides hydrophobicity to the material.

On the other side of the spectrum, adsorbent materials, such as meshes, films, membranes, and clays, are typically used in industrial settings as filters. For instance, meshes are able to adsorb high volumes of oil due to their larger pore sizes. They are usually made from stainless steel or aluminum, and can either be manufactured by, for example, a low-cost 3D printing method or attained commercially. When addressing separation efficiencies, meshes are capable of separating oil/water mixtures with an effectiveness of around 95 to 99%, also being capable of reusage for up to 40 separation cycles. The biggest disadvantage is its inefficiency in collecting oil from an oil/water emulsion, due to their larger pore size, when compared to the oil microdroplets sizes. Clays, such as montmorillonite and bentonite are materials with a high potential for oily wastewater separation purposes. They have not received much attention yet, however, they have been shown to have oil adsorption capacities up to 160 times their own weight and to improve the composite hydrophobicity and physical properties, such as mechanical strength. Even for oil/water emulsion separations, the efficiency reached up to 98%. Textiles possess higher flexibility compared to meshes, and good mechanical strengths. Regardless, they have to be functionalized in order to possess selectivity for oil/water separation, which can reach efficiency values up to 97%. Finally, membranes are generally easy to produce and are cost effective. Additionally, unlike meshes, their ability to achieve small pore sizes makes them suitable to separate oil/water emulsions, presenting efficiencies of around 99% for oil/water mixtures and 97% for oil/water emulsions, although they have a higher tendency to experience membrane fouling. Nanofibrous membranes can be used as a valuable solution to overcome fouling, because of its high surface areas, along with their easy manipulation and customization of their wetting properties, their fiber sizes, and membrane pore sizes, mainly when produced by electrospinning. Membranes can be divided into ceramic membranes, such as silica, and alumina, or be titania-based being more expensive than polymer-based ones; however, having higher temperature resistance. Carbon membranes, based on carbon nanotubes or graphene, help increase water flux and have the ability to adjust their pore sizes. Polymeric membranes are more cost-effective and easier to produce, although they have a higher tendency to deteriorate or to experience membrane fouling. Roughly, membranes can present separation efficiencies of almost up to 99.99% to water/oil mixtures and between 83 to 99.97% to oil/water emulsions. Their biggest and most valuable advantages are their ability to be produced by making use of a wide range of materials, the fact that a large number of them are ecofriendly, and, lastly, their ability to be produced at a low-cost and in a simple manner. They also can incorporate nanoparticles, clays, or different materials, with the possibility of achieving optimized composite membranes with complementary properties, making them a valuable option for oily wastewater cleaning in industrial settings.

Although we can see a big evolution in the development of materials that can be used for oil/water separation, there still exist potential areas for the development of materials with enhanced performance in cleaning oily waters. One of the most important areas is concerned with the separation of oil/water emulsions. This type of emulsion is constituted by small droplets of oil dispersed in water and, in many cases, had its origins in accidental oil spills in open waters where the ocean waves break the oil into microdroplets. The fact that the oil microdroplets are invisible to the naked eye and harmful to the food chain makes the removal of the dispersed oil as important as it is challenging. This is a crucial issue, and new advances are expected in this field of research.

Another important question regarding oil/water separation is the prevention of membrane fouling, a phenomenon that decreases the efficiency of a membrane due to oil that is accumulated in the membrane and blocks the water flow. Since the wettability of the material to oil and water that constitute the membranes influences the efficiency of the oil removal, membranes with enhanced oleophobicity are required. In this area, the incorporation of nanoparticles, with an emphasis on the application of clay nanoparticles, represents a way to control the wettability of the surfaces used for oil/water separation.

Another challenge to oil/water separation is directly linked with the cleaning of the materials used for oil removal application, since this cleaning step must be economically viable for the oil and the oil-removal materials to be reused.

Considering all the recent developments and the importance that the water/oil separation has to the environment, it is expected that the use of oleophobic materials become more common in the future.

## Figures and Tables

**Table 1:** Oil removal efficiency for oil/water mixture separation.

	Efficiency for Oil/Water Mixture Separation
Foams and sponges	
Commercial copper foam surface modified with polydopamine, AgNO_3_, n-dodecyl mercaptan	95% for 30 cycles [42]
Commercial polyurethane (PU) sponge with magnetic properties due to a Fe_3_O_4_ nanoparticles coating	35 times its weight [43]
Polymer-based graphene foam (PGB) by a low-cost self-assembly technique of graphene sheets on a PU skeleton	90% for 300 cycles [44]
Aerogels	
A sponge-like aerogel produced by a sol-gel method using organoalkoxysilanes precursors	Absorption capacities up to 10 cycles [48]
Hydrophobic BiOBr-silicone aerogel, fabricated by a sol-gel method	Superhydrophobic aerogels with excellent mechanical properties, superhydrophobicity and degradation stability.
Nanoparticle incorporation	
Polyurethane sponge coated by dip-coating method with polyfluorowax and hydrophobic silica nanoparticles (HPS)	Absorption capacity up to 10 cycles [50]
Polyurethane foam functionalized with magnetic Fe_3_O_4_ superhydrophobic nanoparticles	70 g/g up to 200 cycles, magnetic behavior [50]
Meshes	
Superoleophobic stainless steel mesh coated with TiO_2_ nanoparticles	99% for 40 cycles [52]
Stainless steel mesh coated with the MnO_2_ nanocrystals by hydrothermal synthesis	95.6% [55]
3D printed cellulose acetate meshes	95% [51]
Clays	
Hydrophobic and oleophilic electrospun polyacrylonitrile (PAN) membrane with Cloisite 30B incorporated onto the nanofibers	180 g/g [57]
Textiles	
70% cotton and 30% polyester immersed in a SiO_2_/PS suspension	97% [64]
Membranes	
electrospun polytetrafluoroethylene (PTFE)	99% [92]
poly(arylene ether nitrile) (PEN) nanofibrous membrane, produced by electrospinning and hot-pressing techniques	99%, after 24 h [88]
electrospun polysulfone (PSf) membrane immersed in a NaOH solution	99.99% [36]
electrospun cellulose acetate nanofibrous membrane	99% for more than one cycle [95]

**Table 2:** Oil removal efficiency for oil/water emulsion separation.

	Efficiency for Oil/Water Emulsion Separation
Foams and sponges	
Polyurethane (PU) foam coated in an alkaline medium containing dopamine, dodecanethiol, and fly ash	93% for 15 cycles [107]
Commercial Ti foam treated with a one-step femtosecond laser	99% [108]
Commercial titanium (Ti) foam anodized in non-toxic fluorine-containing electrolyte	99% [109]
Compressed foam fabricated by carbonization of a 3D, commercially available melamine foam	Higher than 98% [110]
Aerogels	
SiO_2_ and polyacrylonitrile (PAN) with the incorporation of SiO_2_ nanoparticles	99% [111]
Aerogel derived from wastepaper and banana peels	99.6% [112]
Chitosan and agarose-based aerogel	99% [113]
Membranes	
Al_2_O_3_ membrane modified with a ZrO_2_ coating, by hydrolysis of ZrCl_4_	97.8% [73]
SiO_2_ nanocomposite polyvinylidene fluoride membrane (PVDF)	98% for10 cycles [116]
Polymer-based nanofiber membrane dip-coated in a solution containing a polymer of intrinsic microporosity (PIM-1)	99.95% for 30 cycles [117]
Polymer-based nanofiber membrane dip-coated in a solution containing a polymer of intrinsic microporosity fluorinated al-kylsilane (PTES)	99.97% for 30 cycles [117]
Electrospun composite membrane with cellulose nanocrystals (CNC) stamped by screen-printing	83%
Electrospun Polyvinylidene fluoride (PVDF) nanofibrous membrane with Triethylamine	99% [118]
Clays	
Brazilian clays (green calcium bentonite−aluminum clay minerals) modified with surfactants into their interlayer space	96% [120]
Attapulgite and poly(vinyl alcohol) (PVA) nanofibrous membrane via papermaking and posterior sintering	97% [122]
Polymer foam prepared by dip coating in silanized clay nanotube dispersion	105 times its weight in chloroform for 10 cycles [124]

## Data Availability

Not applicable.

## References

[1] Bithas K., Kalimeris P. (2016). Revisiting the Energy-Development Link.

[2] Lee C.H., Tiwari B., Zhang D., Yap Y.K. (2017). Water purification: Oil–water separation by nanotechnology and environmental concerns. Environ. Sci. Nano.

[3] Peterson C.H., Anderson S.S., Cherr G.N., Ambrose R.F., Anghera S., Bay S., Blum M., Condon R., Dean T.A., Graham M. (2012). A Tale of Two Spills: Novel Science and Policy Implications of an Emerging New Oil Spill Model. Bioscience.

[4] Căprărescu S., Modrogan C., Purcar V., Dăncilă A.M., Orbuleț O.D. (2021). Study of Polyvinyl Alcohol-SiO_2_ Nanoparticles Polymeric Membrane in Wastewater Treatment Containing Zinc Ions. Polymers.

[5] Zahid M., Rashid A., Akram S., Rehan Z.A., Razzaq W. (2018). A Comprehensive Review on Polymeric Nano-Composite Membranes for Water Treatment. J. Membr. Sci. Technol..

[6] Ritchie H., Roser M., Rosado P. “Energy,” OurWorldInData.org. https://ourworldindata.org/energy.

[7] Huang J., Wang S., Lyu S., Fu F. (2018). Preparation of a robust cellulose nanocrystal superhydrophobic coating for self-cleaning and oil-water separation only by spraying. Ind. Crops Prod..

[8] Yu L., Han M., He F. (2017). A review of treating oily wastewater. Arab. J. Chem..

[9] Almeida A.P.C., Oliveira J., Fernandes S.N., Godinho M.H., Canejo J.P. (2020). All-cellulose composite membranes for oil microdroplet collection. Cellulose.

[10] Zhan H., Peng N., Lei X., Huang Y., Li D., Tao R., Chang C. (2018). UV-induced self-cleanable TiO2/nanocellulose membrane for selective separation of oil/water emulsion. Carbohydr. Polym..

[11] Xu H., Liu J., Wang Y., Cheng G., Deng X., Li X. (2014). Oil removing efficiency in oil–water separation flotation column. Desalin. Water Treat..

[12] Haddada R., Ferjani E., Roudesli M.S., Deratani A. (2004). Properties of cellulose acetate nanofiltration membranes. Application to brackish water desalination. Desalination.

[13] Frising T., Noïk C., Dalmazzone C. (2006). The Liquid/Liquid Sedimentation Process: From Droplet Coalescence to Technologically Enhanced Water/Oil Emulsion Gravity Separators: A Review. J. Dispers. Sci. Technol..

[14] Jiang J., Zhang Q., Zhan X., Chen F. (2019). A multifunctional gelatin-based aerogel with superior pollutants adsorption, oil/water separation and photocatalytic properties. Chem. Eng. J..

[15] Cambiella A., Benito J.M., Pazos C., Coca J. (2006). Centrifugal Separation Efficiency in the Treatment of Waste Emulsified Oils. Chem. Eng. Res. Des..

[16] Chen W., Su Y., Zheng L., Wang L., Jiang Z. (2009). The improved oil/water separation performance of cellulose acetate-graft-polyacrylonitrile membranes. J. Memb. Sci..

[17] Fakhru’l-Razi A., Pendashteh A., Abdullah L.C., Biak D.R.A., Madaeni S.S., Abidin Z.Z. (2009). Review of technologies for oil and gas produced water treatment. J. Hazard. Mater..

[18] Cheryan M., Rajagopalan N. (1998). Membrane processing of oily streams. Wastewater treatment and waste reduction. J. Memb. Sci..

[19] Song J., Lu Y., Luo J., Huang S., Wang L., Xu W., Parkin I.P. (2015). Barrel-Shaped Oil Skimmer Designed for Collection of Oil from Spills. Adv. Mater. Interfaces.

[20] Mansour L.B., Chalbi S. (2006). Removal of oil from oil/water emulsions using electroflotation process. J. Appl. Electrochem..

[21] Changmai M., Pasawan M., Purkait M.K. (2019). Treatment of oily wastewater from drilling site using electrocoagulation followed by microfiltration. Sep. Purif. Technol..

[22] Butler E., Hung Y.-T., Yeh R.Y.-L., Suleiman Al Ahmad M. (2011). Electrocoagulation in Wastewater Treatment. Water.

[23] Holt P., Barton G., Mitchell C. (1999). Electrocoagulation as a wastewater treatment. Third Annu. Aust. Environ. Eng. Res. Event.

[24] Guerra F., Attia M., Whitehead D., Alexis F. (2018). Nanotechnology for Environmental Remediation: Materials and Applications. Molecules.

[25] Thrall J.H. (2004). Nanotechnology and Medicine. Radiology.

[26] Rae A. (2005). Real life applications of nanotechnology in electronics. IPC—Electronic Circuits World Convention, Printed Circuits Expo, Apex, and the Designers Summit 2005, ECWC 10: The Perfect Fit.

[27] Whitesides G. (2005). Nanoscience, Nanotechnology, and Chemistry. Small.

[28] Singh N.A. (2016). Nanotechnology Definitions, Research, Industry and Property Rights. Nanoscience in Food and Agriculture.

[29] Drexler K.E. (2004). Nanotechnology: From Feynman to Funding. Bull. Sci. Technol. Soc..

[30] Subedi S.K. (2015). An introduction to nanotechnology and its implications. Himal. Phys..

[31] Kargozar S., Mozafari M. (2018). Nanotechnology and Nanomedicine: Start small, think big. Mater. Today Proc..

[32] Khan I., Saeed K., Khan I. (2019). Nanoparticles: Properties, applications and toxicities. Arab. J. Chem..

[33] Atta A., Al-Lohedan H., Al-Hussain S. (2015). Functionalization of Magnetite Nanoparticles as Oil Spill Collector. Int. J. Mol. Sci..

[34] João, Godinho P.H., Godinho M.H. (2019). João Diogo Fidalgo Peça de Oliveira Membranas Celulósicas para Recolha de Microgotas de Óleo. http://hdl.handle.net/10362/77150.

[35] Obaid M., Barakat N.A.M., Fadali O.A., Motlak M., Almajid A.A., Khalil K.A. (2015). Effective and reusable oil/water separation membranes based on modified polysulfone electrospun nanofiber mats. Chem. Eng. J..

[36] Obaid M., Yang E., Kang D.-H., Yoon M.-H., Kim I.S. (2018). Underwater superoleophobic modified polysulfone electrospun membrane with efficient antifouling for ultrafast gravitational oil-water separation. Sep. Purif. Technol..

[37] Ge J., Zhao H.-Y., Zhu H.-W., Huang J., Shi L.-A., Yu S.-H. (2016). Advanced Sorbents for Oil-Spill Cleanup: Recent Advances and Future Perspectives. Adv. Mater..

[38] Gupta R.K., Dunderdale G.J., England M.W., Hozumi A. (2017). Oil/water separation techniques: A review of recent progresses and future directions. J. Mater. Chem. A.

[39] Zhang X., Li Z., Liu K., Jiang L. (2013). Bioinspired Multifunctional Foam with Self-Cleaning and Oil/Water Separation. Adv. Funct. Mater..

[40] Jiang S., Agarwal S., Greiner A. (2017). Low-Density Open Cellular Sponges as Functional Materials. Angew. Chemie Int. Ed..

[41] Pinto J., Athanassiou A., Fragouli D. (2018). Surface modification of polymeric foams for oil spills remediation. J. Environ. Manag..

[42] Zhou W., Li G., Wang L., Chen Z., Lin Y. (2017). A facile method for the fabrication of a superhydrophobic polydopamine-coated copper foam for oil/water separation. Appl. Surf. Sci..

[43] Liu S., Xu Q., Latthe S.S., Gurav A.B., Xing R. (2015). Superhydrophobic/superoleophilic magnetic polyurethane sponge for oil/water separation. RSC Adv..

[44] Wu C., Huang X., Wu X., Qian R., Jiang P. (2013). Mechanically Flexible and Multifunctional Polymer-Based Graphene Foams for Elastic Conductors and Oil-Water Separators. Adv. Mater..

[45] He Z., Zhang X., Batchelor W. (2016). Cellulose nanofibre aerogel filter with tuneable pore structure for oil/water separation and recovery. RSC Adv..

[46] Hench L.L., West J.K. (1990). The sol-gel process. Chem. Rev..

[47] Purcar V., Şomoghi R., Niţu S., Nicolae C.-A., Alexandrescu E., Gîfu I., Gabor A., Stroescu H., Ianchiş R., Căprărescu S. (2017). The Effect of Different Coupling Agents on Nano-ZnO Materials Obtained via the Sol–Gel Process. Nanomaterials.

[48] Yu Y., Wu X., Fang J. (2015). Superhydrophobic and superoleophilic “sponge-like” aerogels for oil/water separation. J. Mater. Sci..

[49] Ge B., Ren G., Yang H., Yang J., Pu X., Li W., Jin C., Zhang Z. (2020). Fabrication of BiOBr-silicone aerogel photocatalyst in an aqueous system with degradation performance by sol-gel method. Sci. China Technol. Sci..

[50] Ge B., Zhu X., Li Y., Men X., Li P., Zhang Z. (2015). Versatile fabrication of magnetic superhydrophobic foams and application for oil–water separation. Colloids Surfaces A Physicochem. Eng. Asp..

[51] Koh J.J., Lim G.J.H., Zhou X., Zhang X., Ding J., He C. (2019). 3D-Printed Anti-Fouling Cellulose Mesh for Highly Efficient Oil/Water Separation Applications. ACS Appl. Mater. Interfaces.

[52] Li J., Yan L., Hu W., Li D., Zha F., Lei Z. (2016). Facile fabrication of underwater superoleophobic TiO_2_ coated mesh for highly efficient oil/water separation. Colloids Surfaces A Physicochem. Eng. Asp..

[53] Milionis A., Bayer I.S., Loth E. (2016). Recent advances in oil-repellent surfaces. Int. Mater. Rev..

[54] Ou R., Wei J., Jiang L., Simon G.P., Wang H. (2016). Robust Thermoresponsive Polymer Composite Membrane with Switchable Superhydrophilicity and Superhydrophobicity for Efficient Oil–Water Separation. Environ. Sci. Technol..

[55] Wang J., Han F., Chen Y., Wang H. (2019). A pair of MnO_2_ nanocrystal coatings with inverse wettability on metal meshes for efficient oil/water separation. Sep. Purif. Technol..

[56] Fischer S., Thümmler K., Volkert B., Hettrich K., Schmidt I., Fischer K. (2008). Properties and Applications of Cellulose Acetate. Macromol. Symp..

[57] Kahraman H.T., Yar A., Avcı A., Pehlivan E. (2018). Preparation of nanoclay incorporated PAN fibers by electrospinning technique and its application for oil and organic solvent absorption. Sep. Sci. Technol..

[58] Kim S.W., Han S.O., Sim I.N., Cheon J.Y., Park W.H. (2015). Fabrication and Characterization of Cellulose Acetate/Montmorillonite Composite Nanofibers by Electrospinning. J. Nanomater..

[59] Wang P., Ma J., Wang Z., Shi F., Liu Q. (2012). Enhanced Separation Performance of PVDF/PVP-g-MMT Nanocomposite Ultrafiltration Membrane Based on the NVP-Grafted Polymerization Modification of Montmorillonite (MMT). Langmuir.

[60] Al-Samhan M., Samuel J., Al-Attar F., Abraham G. (2017). Comparative Effects of MMT Clay Modified with Two Different Cationic Surfactants on the Thermal and Rheological Properties of Polypropylene Nanocomposites. Int. J. Polym. Sci..

[61] Darmanin T., Guittard F. (2014). Recent advances in the potential applications of bioinspired superhydrophobic materials. J. Mater. Chem. A.

[62] Zhang J., Seeger S. (2011). Polyester Materials with Superwetting Silicone Nanofilaments for Oil/Water Separation and Selective Oil Absorption. Adv. Funct. Mater..

[63] Xue C.-H., Ji P.-T., Zhang P., Li Y.-R., Jia S.-T. (2013). Fabrication of superhydrophobic and superoleophilic textiles for oil–water separation. Appl. Surf. Sci..

[64] Zhang X., Geng T., Guo Y., Zhang Z., Zhang P. (2013). Facile fabrication of stable superhydrophobic SiO2/polystyrene coating and separation of liquids with different surface tension. Chem. Eng. J..

[65] Liang B., Zhang G., Zhong Z., Sato T., Hozumi A., Su Z. (2019). Substrate-independent polyzwitterionic coating for oil/water separation membranes. Chem. Eng. J..

[66] Shirazi S., Lin C.-J., Chen D. (2010). Inorganic fouling of pressure-driven membrane processes—A critical review. Desalination.

[67] Chang Q., Zhou J., Wang Y., Liang J., Zhang X., Cerneaux S., Wang X., Zhu Z., Dong Y. (2014). Application of ceramic microfiltration membrane modified by nano-TiO2 coating in separation of a stable oil-in-water emulsion. J. Memb. Sci..

[68] Jin F., Lv W., Zhang C., Li Z., Su R., Qi W., Yang Q.-H., He Z. (2013). High-performance ultrafiltration membranes based on polyethersulfone–graphene oxide composites. RSC Adv..

[69] Park E., Barnett S.M. (2001). Oil/Water Separation Using Nanofiltration Membrane Technology. Sep. Sci. Technol..

[70] Muppalla R., Jewrajka S.K., Reddy A.V.R. (2015). Fouling resistant nanofiltration membranes for the separation of oil–water emulsion and micropollutants from water. Sep. Purif. Technol..

[71] Hirose M., Ito H., Kamiyama Y. (1996). Effect of skin layer surface structures on the flux behaviour of RO membranes. J. Memb. Sci..

[72] Padaki M., Surya Murali R., Abdullah M.S., Misdan N., Moslehyani A., Kassim M.A., Hilal N., Ismail A.F. (2015). Membrane technology enhancement in oil–water separation. A review. Desalination.

[73] Zhou J., Chang Q., Wang Y., Wang J., Meng G. (2010). Separation of stable oil–water emulsion by the hydrophilic nano-sized ZrO_2_ modified Al_2_O_3_ microfiltration membrane. Sep. Purif. Technol..

[74] Bruggen B.V.D., Vandecasteele C., Gestel T.V., Doyenb W., Leysenb R. (2003). Review of Pressure-Driven Membrane Processes. Environ. Prog..

[75] Almetwally A.A., El-Sakhawy M., Elshakankery M.H., Kasem M.H. (2017). Technology of nano-fibers: Production techniques and properties—Critical review. J. Text. Assoc..

[76] Lam Z., Anlauf H., Nirschl H. (2020). High-Pressure Jet Cleaning of Polymeric Microfiltration Membranes. Chem. Eng. Technol..

[77] Jiang Y., Hou J., Xu J., Shan B. (2017). Switchable oil/water separation with efficient and robust Janus nanofiber membranes. Carbon N. Y..

[78] Wang X., Cheng W., Wang D., Ni X., Han G. (2019). Electrospun polyvinylidene fluoride-based fibrous nanocomposite membranes reinforced by cellulose nanocrystals for efficient separation of water-in-oil emulsions. J. Memb. Sci..

[79] Alghoraibi I., Alomari S., Barhoum A., Bechelany M., Makhlouf A. (2018). Different Methods for Nanofiber Design and Fabrication. Handbook of Nanofibers.

[80] Bhardwaj N., Kundu S.C. (2010). Electrospinning: A fascinating fiber fabrication technique. Biotechnol. Adv..

[81] Arslan O., Aytac Z., Uyar T. (2016). Superhydrophobic, Hybrid, Electrospun Cellulose Acetate Nanofibrous Mats for Oil/Water Separation by Tailored Surface Modification. ACS Appl. Mater. Interfaces.

[82] Hart E. (1967). Theory of the tensile test. Acta Metall..

[83] Wang X., Yu J., Sun G., Ding B. (2016). Electrospun nanofibrous materials: A versatile medium for effective oil/water separation. Mater. Today.

[84] Chigome S., Darko G., Torto N. (2011). Electrospun nanofibers as sorbent material for solid phase extraction. Analyst.

[85] Persano L., Camposeo A., Pisignano D. (2015). Active polymer nanofibers for photonics, electronics, energy generation and micromechanics. Prog. Polym. Sci..

[86] Kenry, Lim C.T. (2017). Nanofiber technology: Current status and emerging developments. Prog. Polym. Sci..

[87] Agarwal S., Greiner A., Wendorff J.H. (2013). Functional materials by electrospinning of polymers. Prog. Polym. Sci..

[88] He S., Zhan Y., Bai Y., Hu J., Li Y., Zhang G., Zhao S. (2019). Gravity-driven and high flux super-hydrophobic/super-oleophilic poly(arylene ether nitrile) nanofibrous composite membranes for efficient water-in-oil emulsions separation in harsh environments. Compos. Part B Eng..

[89] Jiang T., Carbone E.J., Lo K.W.H., Laurencin C.T. (2015). Electrospinning of polymer nanofibers for tissue regeneration. Prog. Polym. Sci..

[90] Greiner A., Wendorff J.H. (2007). Electrospinning: A fascinating method for the preparation of ultrathin fibers. Angew. Chemie—Int. Ed..

[91] Costa R.G.F., Oliveira J.E., Paula G.F., Picciani P.H.S., Medeiros E.S., Ribeiro C., Mattoso L.H.C. (2012). Eletrofiação de Polímeros em Solução: Parte I: Fundamentação TeÃ^3^rica. Polímeros.

[92] Patel M., Patel J., Pawar Y., Patel N., Shah M. (2020). Membrane-based downhole oil–water separation (DOWS) technology: An alternative to hydrocyclone-based DOWS. J. Pet. Explor. Prod. Technol..

[93] Si Y., Yan C., Hong F., Yu J., Ding B. (2015). A general strategy for fabricating flexible magnetic silica nanofibrous membranes with multifunctionality. Chem. Commun..

[94] Zhang J., Zhang F., Song J., Liu L., Si Y., Yu J., Ding B. (2019). Electrospun flexible nanofibrous membranes for oil/water separation. J. Mater. Chem. A.

[95] Hong S.K., Bae S., Jeon H., Kim M., Cho S.J., Lim G. (2018). An underwater superoleophobic nanofibrous cellulosic membrane for oil/water separation with high separation flux and high chemical stability. Nanoscale.

[96] Benito J.M., Conesa A., Rubio F., Rodríguez M.A. (2005). Preparation and characterization of tubular ceramic membranes for treatment of oil emulsions. J. Eur. Ceram. Soc..

[97] Soares L.G., Alves A.K. (2013). Obtenção por Electrospinning e Caracterização de Fibras Nanoestruturadas de TiO2 e sua Aplicação Fotocatalítica. Digital Repository. http://hdl.handle.net/10183/85045.

[98] Zhang R., Wang X., Song J., Si Y., Zhuang X., Yu J., Ding B. (2015). In situ synthesis of flexible hierarchical TiO 2 nanofibrous membranes with enhanced photocatalytic activity. J. Mater. Chem. A.

[99] Barbosa A.S., Barbosa A.S., Rodrigues M.G.F. (2015). Synthesis of zeolite membrane (MCM-22/α-alumina) and its application in the process of oil-water separation. Desalin. Water Treat..

[100] Al-anzi B.S., Siang O.C. (2017). Recent developments of carbon based nanomaterials and membranes for oily wastewater treatment. RSC Adv..

[101] Elimelech M., Phillip W.A. (2011). The Future of Seawater Desalination: Energy, Technology, and the Environment. Science.

[102] Prince J.A., Bhuvana S., Anbharasi V., Ayyanar N., Boodhoo K.V.K., Singh G. (2016). Ultra-wetting graphene-based PES ultrafiltration membrane—A novel approach for successful oil-water separation. Water Res..

[103] Li F., Bhushan B., Pan Y., Zhao X. (2019). Bioinspired superoleophobic/superhydrophilic functionalized cotton for efficient separation of immiscible oil-water mixtures and oil-water emulsions. J. Colloid Interface Sci..

[104] Meng T., Xie R., Ju X.-J., Cheng C.-J., Wang S., Li P.-F., Liang B., Chu L.-Y. (2013). Nano-structure construction of porous membranes by depositing nanoparticles for enhanced surface wettability. J. Memb. Sci..

[105] Chen C., Weng D., Mahmood A., Chen S., Wang J. (2019). Separation Mechanism and Construction of Surfaces with Special Wettability for Oil/Water Separation. ACS Appl. Mater. Interfaces.

[106] Xue Z., Cao Y., Liu N., Feng L., Jiang L. (2014). Special wettable materials for oil/water separation. J. Mater. Chem. A.

[107] Wang J., Wang H., Geng G. (2018). Flame-retardant superhydrophobic coating derived from fly ash on polymeric foam for efficient oil/corrosive water and emulsion separation. J. Colloid Interface Sci..

[108] Yang S., Yin K., Wu J., Wu Z., Chu D., He J., Duan J.-A. (2019). Ultrafast nano-structuring of superwetting Ti foam with robust antifouling and stability towards efficient oil-in-water emulsion separation. Nanoscale.

[109] Luo Z.-Y., Lyu S.-S., Wang Y.-Q., Mo D.-C. (2017). Fluorine-Induced Superhydrophilic Ti Foam with Surface Nanocavities for Effective Oil-in-Water Emulsion Separation. Ind. Eng. Chem. Res..

[110] Yang S., Chen L., Liu S., Hou W., Zhu J., Zhang Q., Zhao P. (2020). Robust Bifunctional Compressed Carbon Foam for Highly Effective Oil/Water Emulsion Separation. ACS Appl. Mater. Interfaces.

[111] Si Y., Fu Q., Wang X., Zhu J., Yu J., Sun G., Ding B. (2015). Superelastic and Superhydrophobic Nanofiber-Assembled Cellular Aerogels for Effective Separation of Oil/Water Emulsions. ACS Nano.

[112] Yue X., Zhang T., Yang D., Qiu F., Li Z. (2018). Hybrid aerogels derived from banana peel and waste paper for efficient oil absorption and emulsion separation. J. Clean. Prod..

[113] Chaudhary J.P., Vadodariya N., Nataraj S.K., Meena R. (2015). Chitosan-Based Aerogel Membrane for Robust Oil-in-Water Emulsion Separation. ACS Appl. Mater. Interfaces.

[114] Ma W., Zhang Q., Hua D., Xiong R., Zhao J., Rao W., Huang S., Zhan X., Chen F., Huang C. (2016). Electrospun fibers for oil–water separation. RSC Adv..

[115] Zarghami S., Mohammadi T., Sadrzadeh M., Van der Bruggen B. (2019). Superhydrophilic and underwater superoleophobic membranes—A review of synthesis methods. Prog. Polym. Sci..

[116] Cui J., Zhou Z., Xie A., Meng M., Cui Y., Liu S., Lu J., Zhou S., Yan Y., Dong H. (2019). Bio-inspired fabrication of superhydrophilic nanocomposite membrane based on surface modification of SiO2 anchored by polydopamine towards effective oil-water emulsions separation. Sep. Purif. Technol..

[117] Zhang C., Li P., Cao B. (2016). Fabrication of Superhydrophobic–Superoleophilic Fabrics by an Etching and Dip-Coating Two-Step Method for Oil–Water Separation. Ind. Eng. Chem. Res..

[118] Obaid M., Mohamed H.O., Yasin A.S., Yassin M.A., Fadali O.A., Kim H., Barakat N.A.M. (2017). Under-oil superhydrophilic wetted PVDF electrospun modified membrane for continuous gravitational oil/water separation with outstanding flux. Water Res..

[119] Monticelli O., Bottino A., Scandale I., Capannelli G., Russo S. (2007). Preparation and properties of polysulfone–clay composite membranes. J. Appl. Polym. Sci..

[120] Mota M.F., Rodrigues M.G.F., Machado F. (2014). Oil–water separation process with organoclays: A comparative analysis. Appl. Clay Sci..

[121] Moazed H., Viraraghavan T. (2005). Use of Organo-Clay/Anthracite Mixture in the Separation of Oil from Oily Waters. Energy Sources.

[122] Zhu Y., Chen D. (2017). Novel clay-based nanofibrous membranes for effective oil/water emulsion separation. Ceram. Int..

[123] Yu T., Swientoniewski L.T., Omarova M., Li M.-C., Negulescu I.I., Jiang N., Darvish O.A., Panchal A., Blake D.A., Wu Q. (2019). Investigation of Amphiphilic Polypeptoid-Functionalized Halloysite Nanotubes as Emulsion Stabilizer for Oil Spill Remediation. ACS Appl. Mater. Interfaces.

[124] Wu F., Pickett K., Panchal A., Liu M., Lvov Y. (2019). Superhydrophobic Polyurethane Foam Coated with Polysiloxane-Modified Clay Nanotubes for Efficient and Recyclable Oil Absorption. ACS Appl. Mater. Interfaces.

[125] Stehl D., Skale T., Hohl L., Lvov Y., Koetz J., Kraume M., Drews A., von Klitzing R. (2020). Oilc-in-Water Pickering Emulsions Stabilized by Halloysite Clay Nanotubes Toward Efficient Filterability. ACS Appl. Nano Mater..

[126] Panchal A., Swientoniewski L.T., Omarova M., Yu T., Zhang D., Blake D.A., John V., Lvov Y.M. (2018). Bacterial proliferation on clay nanotube Pickering emulsions for oil spill bioremediation. Colloids Surfaces B Biointerfaces.

